# Bibliometric Analysis of Birt-Hogg-Dubé Syndrome From 2001 to 2021

**DOI:** 10.3389/fmed.2022.857127

**Published:** 2022-04-11

**Authors:** Shixu Liu, Kun Xia, Xiaohong Liu, Yuanyuan Duan, Mu Hu, Hongsheng Xia, Jiayu Lv, Lili Zhang, Yanyi Liu, Xiao Xia, Guangxi Li, Xiangning Cui

**Affiliations:** ^1^Guanganmen Hospital, China Academy of Chinese Medical Sciences, Beijing, China; ^2^Graduate School of China Academy of Chinese Medical Sciences, Beijing, China; ^3^The First Affiliated Hospital of Guangzhou University of Chinese Medicine, Guangzhou, China; ^4^Graduate School of Beijing University of Chinese Medicine, Beijing, China

**Keywords:** Birt-Hogg-Dubé syndrome (BHD), *FLCN*, folliculin, bibliometric analysis, CiteSpace, VOSviewer

## Abstract

**Background:**

Birt-Hogg-Dubé syndrome (BHD) is a rare autosomal dominant inherited disorder caused by germline mutations in *folliculin (FLCN)*. Despite our significantly evolved understanding of BHD over the past decades, no bibliometric analyses have been conducted in this field. This study aimed to analyze and visualize the characteristics of publication outputs, the research hotspots, and scientific frontiers about BHD using bibliometric analysis.

**Methods:**

All relevant literature on BHD was culled from the Web of Science Core Collection (WoSCC) database. Valid data were extracted from the articles and visually analyzed using CiteSpace and VOSviewer.

**Results:**

A total of 751 qualifying papers were included. Publication outputs concerning BHD increased over time. The dominant position of the United States and Japan in BHD research field was evident. National Cancer Institute (the USA) and Yokohama City University (Japan) were the two most productive organizations. W. Marston Linehan exerted a considerable publication impact and had made the most remarkable contributions in the field of BHD. *Plos One* was the journal with the highest publication outputs, and half of the top 10 journals and co-cited journals belonged to Q1 or Q2. Keyword citation bursts revealed that management, tumor suppressor, *flcn* gene, spectrum, diagnosis, risk, computed tomography were the emerging research hotspots.

**Conclusion:**

Research on BHD is prosperous. International cooperation between countries and organizations is also expected to deepen and strengthen in the future. Our results indicated that *FLCN*-associated pathways involved in the pathogenesis of BHD, specific options for early diagnosis, and molecular-targeting therapies will remain research hotspots in the future.

## Introduction

Birt-Hogg-Dubé syndrome (BHD, OMIM#135150) is an inherited autosomal dominant disorder that may involve multiple organs including the skin, the lung, and the kidney. This syndrome was actually first described in 1975 by Otto P. Hornstein and Monika Knickenberg from Germany. They pointed out that the occurrence of multiple “perifollicularfibromas” represented an autosomal dominant trait indicating extracutaneous cancer proneness (1). Two years later, Arthur R.Birt, Georgina R. Hogg, and W. James Dubé delineated similar hereditary skin lesions for which they proposed the name “fibrofolliculoma”, without mentioning extracutaneous cancer proneness (2). Then, the disease was named “Birt-Hogg-Dubé syndrome” after the three Canadian physicians. Nowadays, many scholars believe that “fibrofolliculoma” is identical to “perifollicular fibroma” (3). Thus, these two diseases are basically the same. Although BHD is more widely accepted, we should not forget the persons who first discovered the disease.

As a rare syndrome, its exact incidence is unknown (with only 600 families reported worldwide) (4). BHD is one of the three major reasons for cystic lung disease with lymphangioleiomyomatosis (LAM) and pulmonary Langerhans cell histiocytosis (PLCH). Unlike LAM, BHD has no gender predilection (5) and shows a tendency to affect patients in their thirties to forties (6). In 2001, a BHD-related gene locus was located to chromosome 17p11.2 by linkage analysis (7, 8). One year later, germline mutations in *FLCN* gene, encoding the protein folliculin, was identified as the causative gene of BHD (9).

The clinical expression of BHD typically includes cutaneous fibrofolliculomas, multiple pulmonary cysts, recurrent spontaneous pneumothorax, and renal tumors of various histological types (10). Early diagnosis of the syndrome, prompt management for probands and affected family members, early detection and therapeutics of renal cancer, and prevention of pneumothorax are the cornerstones in the management of BHD (10, 11). A diagnostic algorithm proposed by Menko et.al. is listed in Table 1. Notwithstanding remarkable progress in our understanding of BHD over the past several decades, comprehensive reports that can benefit researchers to obtain an intuitive overview and reveal trends in the BHD research field are still absent.

**Table 1:** Diagnostic criteria for Birt–Hogg–Dubé syndrome.

**The patient should fulfill one major or two minor criteria for diagnosis**
**Major criteria**
• At least 5 fibrofolliculomas or trichodiscomas, at least 1 histologically confirmed, of adult-onset
• FLCN germline mutation
**Minor criteria**
• Multiple pulmonary cysts, bilateral and basally located, with no other causes, with or without spontaneous primary pneumothorax
• Renal cancer, early-onset (<50 years), or multifocal and/or bilateral, or hybrid chromophobe and oncocytic histology
• One first-degree relative with BHD

Bibliometric analysis is the use of mathematical and statistical methods to analyze large amounts of heterogeneous literature (12). Combining with visualizing processing applications like CiteSpace (13) and VOSviewer (14), bibliometric analysis can not only analyze the contributions of numerous authors, journals, organizations, and countries/regions, but also can predict the hotspots and trends of a specific research field, providing the basis for the future study (15, 16). However, there is still no bibliometric analysis available in BHD research field. In the present study, we aimed at exploring the hotspots and trends in BHD research by analyzing scientific literature from 2001 to 2021, to provide new visions for future scholars, especially for those who have curiosity but are novices in this field.

## Materials and Methods

### Data Collection

All historic achievements were culled from the Web of Science Core Collection database. Editions selected “Science Citation Index Expanded (SCI-EXPANDED)”. The search string was set to TS = (“Birt-Hogg-Dubé Syndrome” OR “BHD” OR “Hornstein–Knickenberg Syndrome”). The publication period was limited from 2001 to 2021. Of various publication types, only articles and review articles written in English were included. Record content chose full record and cited references. Then exported and saved the record as plain text files, and stored in the format of download_txt. The whole process was completed within 1 day (March 6, 2022).

### Data Analysis

Coherent data were imported into CiteSpace and VOSviewer to perform visual analysis. In the atlas, circles stand for countries/regions, organizations, authors, and keywords; lines between circles represent collaboration or co-citation relationships; a spectrum of colors depicts the temporal order of observations.

CiteSpace version 5.8.R3 (Drexel University, Philadelphia, PA, USA) was used to visualize the intercountry and institutional analysis and the author's cooperation. Developed by Chen, Citespace is an application for progressive knowledge domain visualization (17). It is especially suitable for analyzing and visualizing patterns and trends of scientific literature. The main goal of knowledge domain visualization is to identify key points in the development of the research field. CiteSpace provides a visual aid that depicts the hotspots and evolution processes of research and predicts the future trends of the domain intuitively (13, 18).

VOSviewer version 1.6.17 (Leiden University, Leiden, Netherlands) was chosen for drawing the maps of the references and journal co-citation analysis, as well as keyword co-occurrence. Unlike the conventional tools for building and viewing bibliometric networks, VOSviewer focuses on the graphic representation of knowledge mappings. It is particularly useful to display sizable bibliometrics in an easy-to-explain way (14).

## Results

### The Trend of Publication Outputs

**Figure 1:**
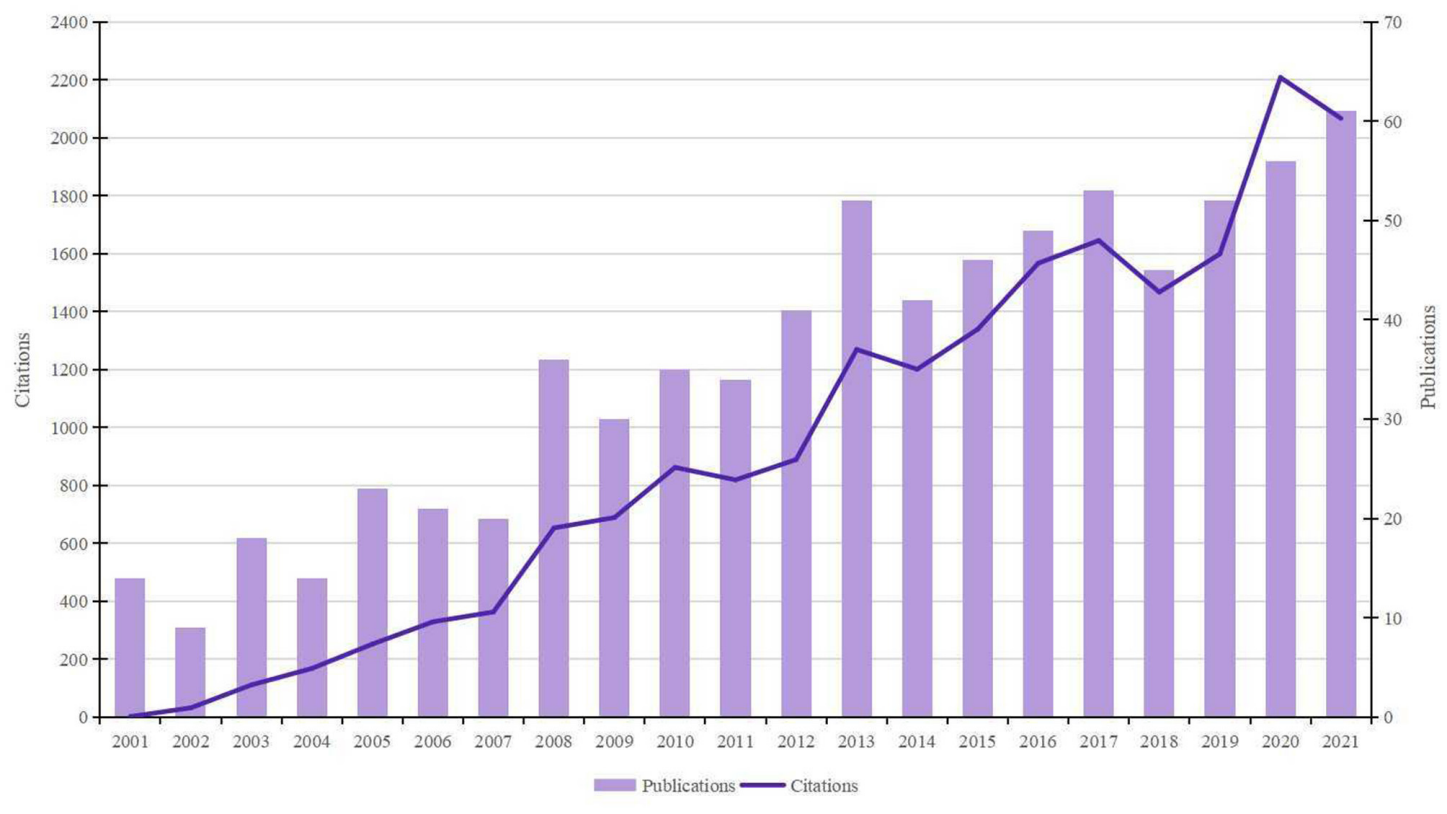
Trends of BHD publications over the past 20 years.

As shown in Figure 1, the number of papers concerning BHD displayed a significant upward trend. A total of 751 publications (650 articles and 101 reviews) met the search strategy. The total citations were 13,604, and the average citations were 26.25 per item. The *h*-index of all papers was 66. 2021 was the peak year of publication outputs with 61 articles.

### Contribution of Countries/Regions and Organizations

**Figure 2:**
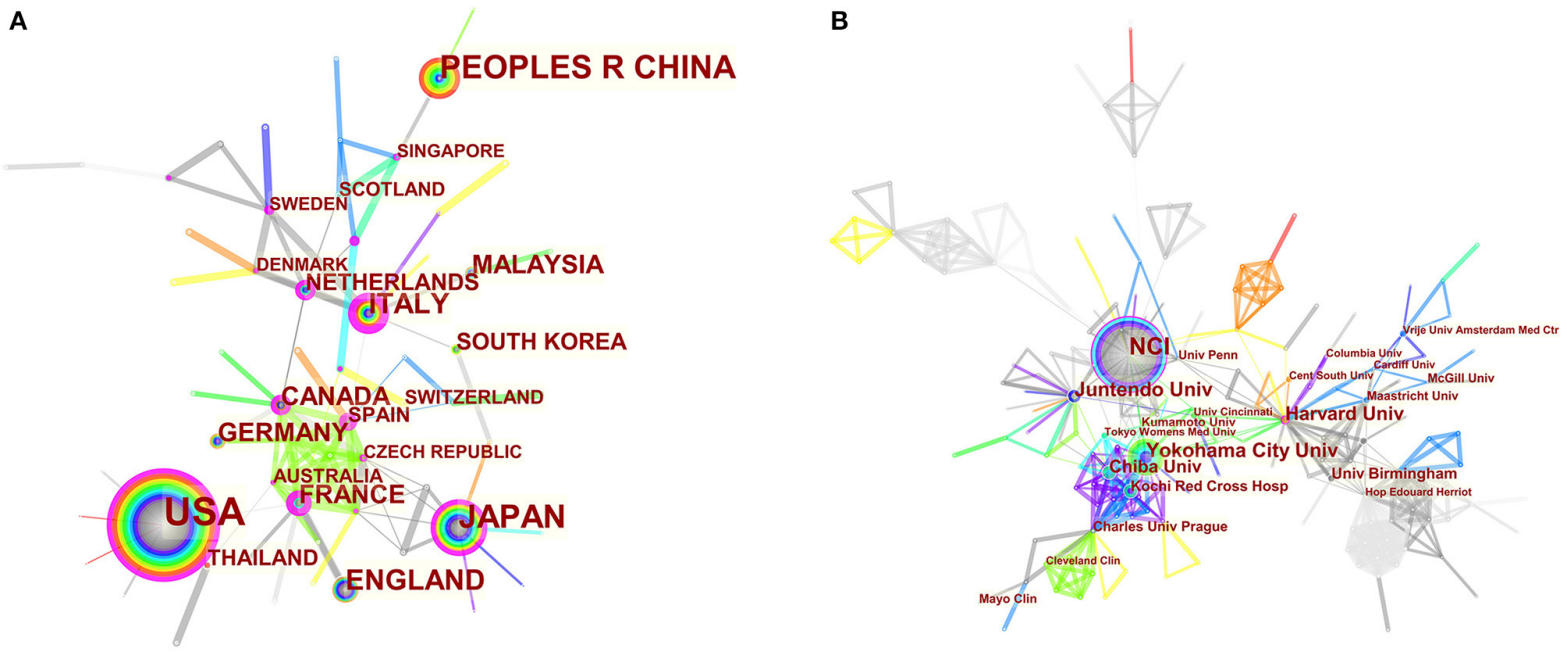
**(A)** CiteSpace network map of countries/regions involved in BHD. **(B)** CiteSpace network map of organizations involved in BHD. Notes: Each circle represents a country/region or organization; the size of the circle is proportional to the publication outputs. The lines between the circles represent the collaboration between countries/regions or organizations; the thicker the lines, the closer the collaboration. The color of a ring denotes a time of publication. The gray ring in the inner circle represents the earliest publications from 2001, and the red ring in the outer circle is the most recent publications. The thickness of a ring is proportional to the number of publications in a given time slice. Purple outer rings indicate a high degree of centrality. Timespan: 2001-2021; Slice length = 1.

The maps of intercountry (Figure 2A) and inter-organizational cooperation (Figure 2B) were plotted using CiteSpace. As shown in Tables 2, 3, we ranked the top 5 most productive countries/regions and organizations in this field. The largest contributor was the United States (*n* = 250, 33.3%), followed by China (*n* = 99, 13.2%), Japan (*n* = 96, 12.8%), England (*n* = 56, 7.5%) and Italy (*n* = 53, 7.1%). The outputs from the two most productive countries were nearly half of the total. Organizations with the highest number of papers were National Cancer Institute (the USA), and Yokohama City University (Japan) ranked second. Countries such as Italy, Canada, France, Spain, the USA, Japan, and China had a high degree of centrality, as indicated by the nodes' purple outer rings in Figure 2A. Centrality is a measure associated with the transformative potential of a scientific contribution. Such nodes tend to bridge different stages of the development of a scientific field (19). There is some active cooperation among countries and organizations, for example, Spain, Canada, Yokohama City University, Chiba University, National Cancer Institute. However, most of them were dispersed and lack intensive cooperation.

**Table 2:** The top 10 countries/regions for publications and centrality in BHD research.

**No**.	**Country**	**Count (%)**	**Country**	**Centrality**
1	USA	250 (33.3%)	France	0.54
2	China	99 (13.2%)	Italy	0.53
3	Japan	96 (12.8%)	Spain	0.52
4	England	56 (7.5%)	Netherlands	0.39
5	Italy	53 (7.1%)	Canada	0.33

**Table 3:** The top 5 institutions for publications and centrality in BHD research.

**No**.	**Institution**	**Count (%)**	**Institution**	**Centrality**
1	National Cancer Institute	57(7.2%)	Harvard Univ	0.14
2	Yokohama City Univ	28(3.7%)	National Cancer Institute	0.1
3	Juntendo Univ	25(3.3%)	Yokohama City Univ	0.07
4	Harvard Univ	24(3.2%)	Juntendo Univ	0.04
5	Chiba Univ	18(2.4%)	Univ Penn	0.04

### Authors and Co-cited Authors

**Figure 3:**
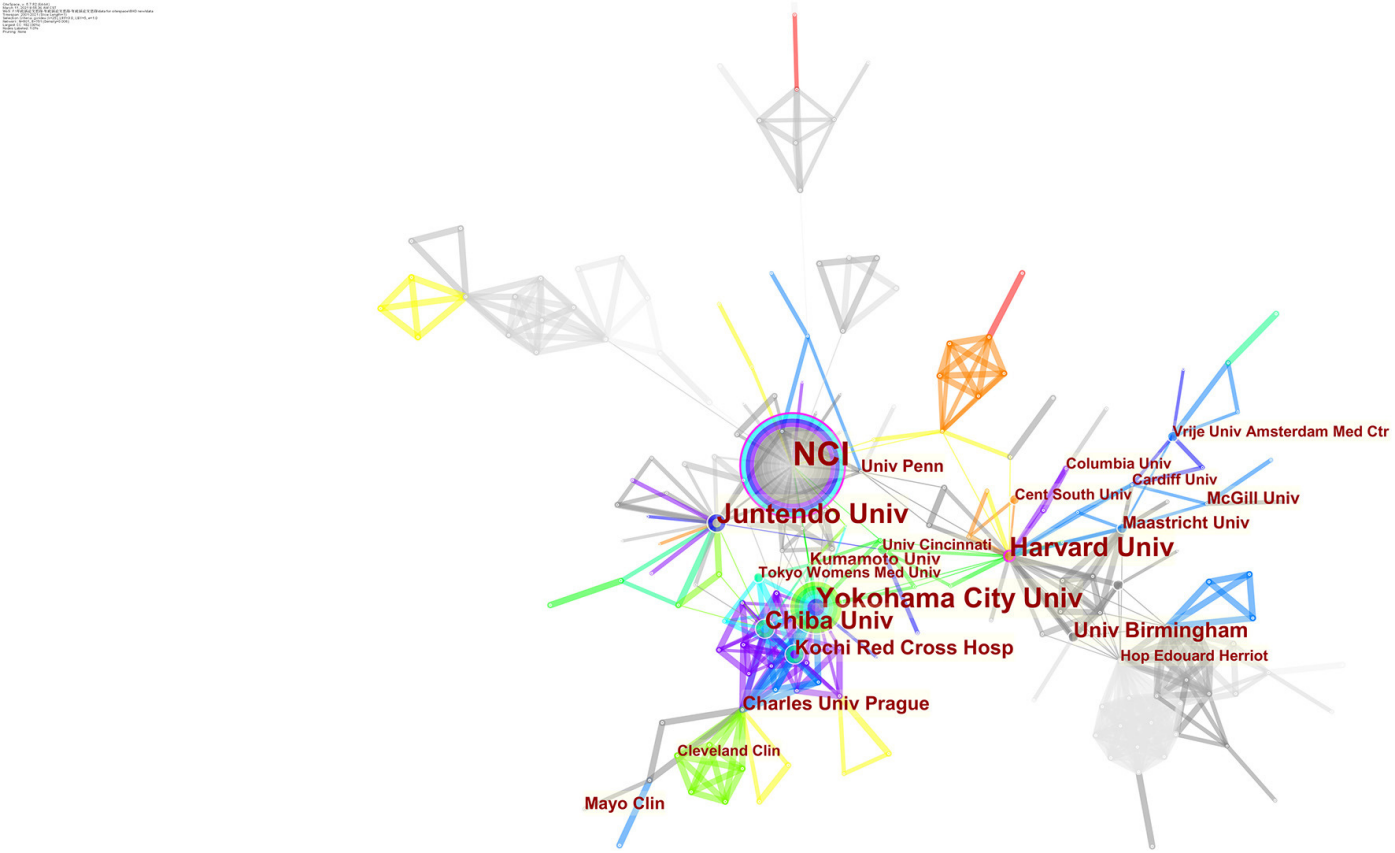
CiteSpace network map of active authors in the field of BHD. Notes: Each circle represents an author. The size of the circle is proportional to the publication outputs, and lines between two circles represent a collaboration between two authors on the same article. Wider lines imply a stronger connection between scholars. Timespan: 2001-2021; Slice length = 1.

Table 4 lists the top 10 most active authors, authors with the highest citations, and co-cited authors. WM Linehan was the most productive scholar with the highest number of publications as well as citations, followed by LS Schmidt. Generally, centrality >0.1 is regarded as relatively essential nodes. However, the highest centrality of authors is 0.04 (M Furuya and JP Gille) and WM Linehan ranked the third (0.03), others are 0.01 or 0. This may be due to the scholars in the BHD research field lacking in cooperation and having a relatively small impact on each others' work. Figure 3 generated by CiteSpace demonstrated the scholars' cooperation relationships. There is an obvious cooperation network between different scholars, for example, WM Linehan, LS Schmidt, M Baba, JR Toro.

**Table 4:** The top 5 authors, cited authors, and co-cited authors in BHD research.

**No**.	**Author**	**Count (%)**	**Centrality**	**Cited author**	**Citations**	**Co-cited author**	**Citations**
1	WM Linehan	53(7.1%)	0.03	WM Linehan	5,416	JR Toro	425
2	LS Schmidt	39(5.2%)	0.01	LS Schmidt	3,910	LS Schmidt	370
3	M Furuya	26(3.5%)	0.04	B Zbar	3,037	M Baba	283
4	M Baba	20(2.7%)	0	M Baba	1,245	ML Nickerson	276
5	B Zbar	20(2.7%)	0.01	O Hes	8,95	B Zbar	256
6	Y Nakatani	17(2.3%)	0	ER Maher	6,59	AR Birt	245
7	K Seyama	17(2.3%)	0.01	FH Menko	6,00	CP Pavlovich	236
8	M Yao	17(2.3%)	0	M Van Stennsel	5,98	SK Khoo	215
9	ER Maher	16(2.1%)	0	H Hasumi	5,31	FH Menko	175
10	M Kurihara	16(2.1%)	0	K Seyama	4,85	H Hasumi	149

Co-cited authors are two or more authors who are cited together by later publications, and they build a co-citation relationship. Eight authors had a citation frequency of more than 200 times. JR Toro (425) was the most frequently cited author, followed by LS Schmidt (370).

### Journals and Co-cited Journals

446 academic journals were involved in the BHD research field. Plos One contributed the majority of articles (19, 2.5%), and Proceedings of the National Academy of Sciences of the United States of America (PNAS) had the highest impact factor among the top 10 journals.

The number of times that an academic journal is co-cited, is one of the indicators to measure whether the journal has notable influence in a domain. Four journals had been co-cited more than 500 times among the top 10 co-cited journals. *PNAS* had the highest level of co-citations (666), followed by *Journal of Medical Genetics* (653). As shown in Table 5, most of the top 10 journals and co-cited journals belong to Q1 or Q2 based on the newest journal citation reports (JCR) in 2020.

**Table 5:** The top 10 journals and co-cited journals related to BHD.

**No**.	**Journal**	**Count (%)**	**IF(2020)**	**JCR**	**Co-cited Journal**	**Citation**	**IF(2020)**	**JCR**
1	Plos One	19(2.5%)	3.240	Q3	Proc Natl Acad Sci USA	666	11.205	Q1
2	Fam Cancer	12(1.6%)	2.375	Q4	J Med Genet	653	6.318	Q1
3	J Med Genet	10(1.3%)	6.318	Q1	Am J Surg Pathol	579	6.394	Q1
4	Oncogene	9(1.2%)	9.867	Q1	J Urol	531	7.450	Q1
5	Orphanet J Rare Dis	9(1.2%)	4.123	Q2	Arch Dermatol	473	NA	NA
6	Am J Roentgenol	8(1.1%)	3.959	Q2	Oncogene	469	9.867	Q1
7	Hum Mol Genet	8(1.1%)	6.150	Q2	Am J Hum Biol	463	1.937	Q4
8	Evid Based Complement Alternat Med	8(1.1%)	2.629	Q4	Cancer Cell	400	31.743	Q2
9	Proc Natl Acad Sci USA	8(1.1%)	11.205	Q1	Am J resp Crit Care	377	21.405	Q1
10	Intern Med	7(0.9%)	1.271	Q4	Plos One	312	3.240	Q3

### Co-cited References and References Burst

**Figure 4:**
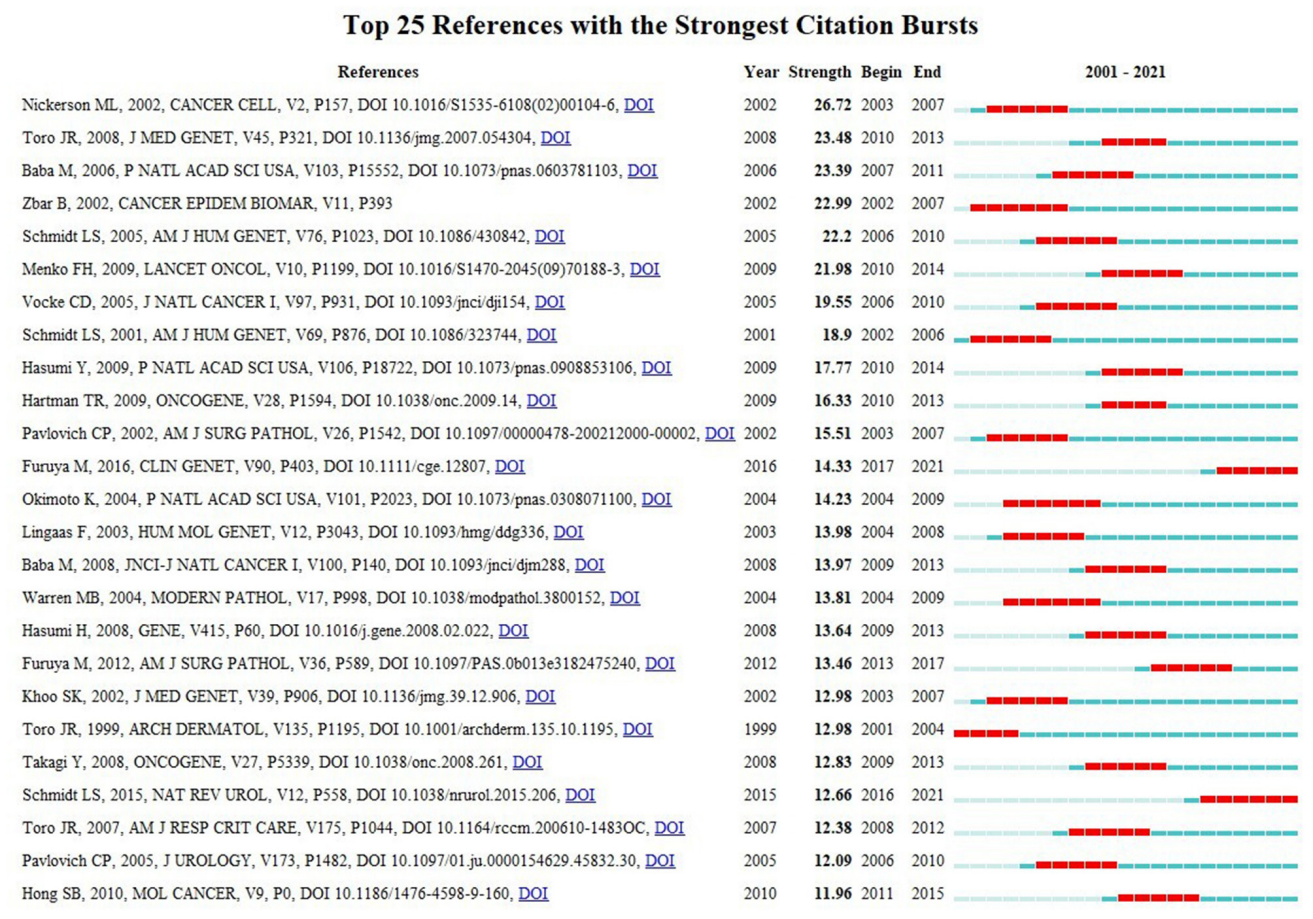
Twenty five references with the strongest citation bursts related to BHD. Notes: This visualization shows which references have the strongest citation bursts and which periods the strongest bursts took place. For example, from the list, we can tell that Nickerson et al. (9) has the strongest bursts among articles published from 2003 to 2007.

Co-citation implies that two or more articles are cited simultaneously by more than one later publication. It is a quantification to measure the degree of relationship between references. Table 6 presents the ten most often co-cited references among the 106 references retrieved. Figure 4 discloses the top 25 references with the strongest citation bursts. It provides evidence that a particular article is associated with a surge of citations. In other words, articles with strong citation bursts are paid much attention by scholars in the scientific community (26). The first paper as ranked by both co-citation and burst strength is published in *Cancer Cell* by ML Nickerson in 2002 (9). The majority of them have been cited frequently during 2001–2021. We can infer that the research concerning BHD may continue to be prosperous in the future.

**Table 6:** The top 10 co-cited references related to BHD.

**No**.	**References**	**Author**	**Year**	**Journal**	**Citations**	**Ref**.
1	Mutations in a novel gene lead to kidney tumors, lung wall defects, and benign tumors of the hair follicle in patients with the Birt-Hogg-Dube syndrome	ML Nickerson	2002	Cancer Cell	266	(9)
2	Hereditary multiple fibrofolliculomas with trichodiscomas and acrochordons	AR Birt	1977	Arch Dermatol	242	(2)
3	Risk of renal and colonic neoplasms and spontaneous pneumothorax in the Birt-Hogg-Dube syndrome	B Zbar	2002	Cancer Epidem Biomar	192	(20)
4	Coupling of STIM1 to store-operated Ca2+ entry through its constitutive and inducible movement in the endoplasmic reticulum	M Baba	2006	P Natl Acad Sci USA	153	(21)
5	BHD mutations, clinical and molecular genetic investigations of Birt–Hogg–Dubé syndrome: a new series of 50 families and a review of published reports	JR Toro	2008	J Med Genet	159	(22)
6	Germline BHD-mutation spectrum and phenotype analysis of a large cohort of families with Birt-Hogg-Dube syndrome	LS Schmidt	2005	Am J Hum Genet	154	(23)
7	Birt-Hogg-Dubé syndrome: diagnosis and management	FH Menko	2009	Lancet Oncol	159	(6)
8	Renal tumors in the Birt-Hogg-Dube syndrome	CP Pavlovich	2002	Am J Surg Pathol	136	(8)
9	Birt-Hogg-Dube syndrome: a novel marker of kidney neoplasia	JR Toro	1999	Arch Dermatol	130	(24)
10	Birt-Hogg-Dube syndrome, a genodermatosis associated with spontaneous pneumothorax and kidney neoplasia, maps to chromosome 17p11. 2	LS Schmidt	2001	Am J Hum Genet	113	(25)

### The Analysis of Hotspots and Frontiers

Research topic and essences are summarized by keywords. High-frequency keywords generally are the dominant research direction. Keyword co-occurrence analysis offers assistance to find research hotspots and predicts research trends in a certain field (17). According to Table 7, the top 10 keywords with the highest frequency in 2001–2021 are Birt-Hogg-Dubé syndrome (347), spontaneous pneumothorax (168), mutation (159), renal cell carcinoma (119), family (83), *folliculin* (83), BHD gene (68), tumor (67), cancer (66), fibrofolliculoma (66).

**Table 7:** Top 20 keywords related to BHD.

**Rank**	**Keywords**	**Count**	**Rank**	**Keywords**	**Count**
1	Birt-Hogg-Dube syndrome	347	11	FLCN	57
2	Spontaneous pneumothorax	168	12	Gene	56
3	Mutation	159	13	Protein	50
4	Renal cell carcinoma	119	14	Pulmonary cyst	40
5	Family	83	15	Diagnosis	39
6	Folliculin	83	16	Kidney	39
7	BHD gene	68	17	Management	36
8	Tumor	67	18	Activation	34
9	Cancer	66	19	Tumor suppressor gene	33
10	Fibrofolliculoma	66	20	Risk	32

**Figure 5:**
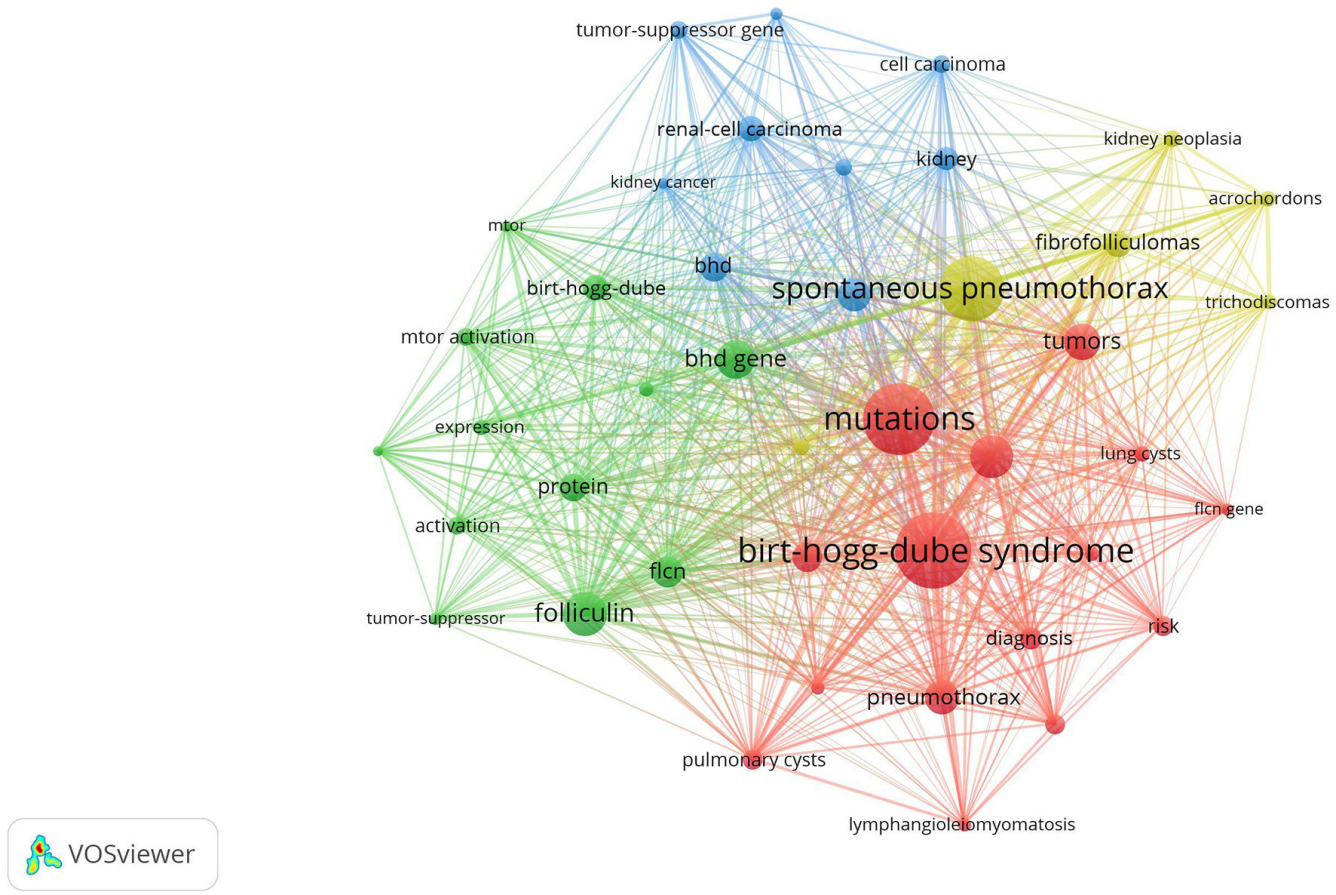
Keywords clustering analysis of the BHD research. Notes: The keywords that occurred more than 20 times were divided into four clusters by different colors: cluster 1 (red), cluster 2 (green), cluster 3 (blue), cluster 4 (yellow). The size of the nodes represents the frequency of occurrences.

Clustered keywords reflect knowledge structure in a particular topic. We use VOSviewer software for this process. Nodes and labels constitute a unit, and units with different colors make up different clusters. There are red, green, blue, and yellow clusters as shown in Figure 5, which respectively represent four research directions. The main keywords of the red cluster are diagnosis, families, mutations, management, pneumothorax, lung cysts, lymphangiomyomatosis. The green cluster covers folliculin, flcn, BHD gene, expression, mTOR activation, identification, protein, polycystic kidneys. The blue cluster mainly includes renal-cell carcinoma, Hippel-Lindau-disease, kidney, kidney cancer, tumor-suppressor gene, and of the yellow cluster are spontaneous pneumothorax acrochordon, fibrofolliculoma, trichodiscoma, kidney neoplasia.

Strong citation bursts can unveil hot words at the frontier of research. Table 8 listed the top 20 keywords with the strongest citation bursts and sorted them by their strength. The top five hotspots were: tumor suppressor gene (10.38), BHD gene (8.52), kidney neoplasia (8.44), *folliculin* (8.16), acrochordon (7.56). Besides, keywords such as “management”, “tumor suppressor”, “*flcn*”, “spectrum”, “diagnosis” turned up constantly during the past 5 years (Table 9).

**Table 8:** Top 20 keywords with the strongest citation bursts.

**Keywords**	**Strength**	**Begin**	**End**	**2001–2021**
Tumor suppressor gene	10.38	2003	2021	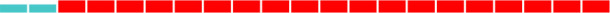
BHD gene	8.52	2005	2021	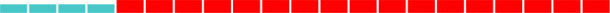
Kidney neoplasia	8.44	2002	2021	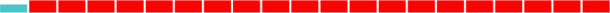
Folliculin	8.16	2013	2021	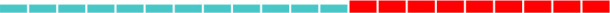
Acrochordon	7.56	2001	2021	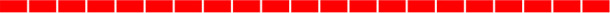
mTOR activation	5.02	2012	2021	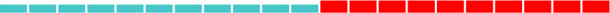
Protein	4.65	2011	2021	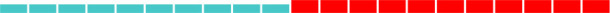
Fumarate hydratase	4.29	2006	2021	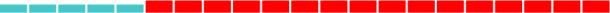
Intestinal polyposis	4.23	2002	2021	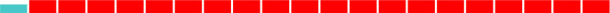
Family	4.23	2010	2021	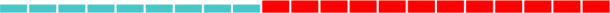
Trichodiscoma	4.16	2001	2021	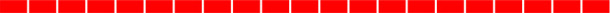
Birt-Hogg-Dube	4.06	2006	2021	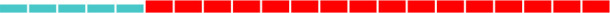
Renal cancer	3.92	2013	2021	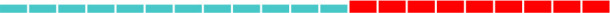
German shepherd dog	3.76	2004	2021	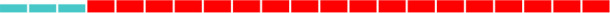
mTOR	3.73	2009	2021	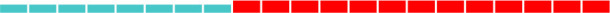
Cell carcinoma	3.36	2002	2021	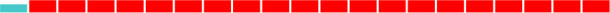
Gene product	3.32	2012	2021	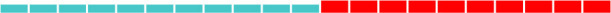
bhd	3.24	2006	2021	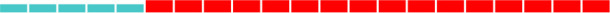
nodular dermatofibrosis	3.22	2004	2021	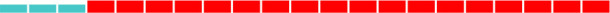
interact	3.14	2013	2021	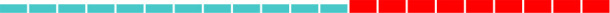

*The beginning of a blue line depicts when an article is published. The beginning of a red segment marks the beginning of a period of burst, whereas the end of the red segment marks the end of the burst period*.

**Table 9:** The strongest citation bursts keywords after 2016.

**Keywords**	**Strength**	**Begin**	**End**	**2016–2021**
Management	5.84	2016	2021	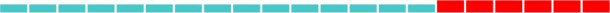
Tumor suppressor	4.63	2016	2021	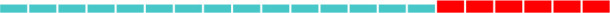
FLCN	3.67	2016	2021	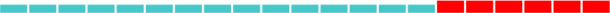
Pneumothorax	3.31	2016	2021	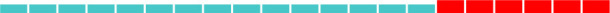
Diagnosis	4.83	2017	2021	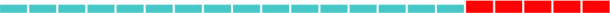
Spectrum	4.7	2017	2021	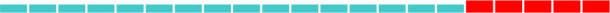
FLCN gene	4.38	2017	2021	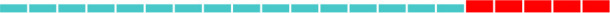
Kidney cancer	3.31	2017	2021	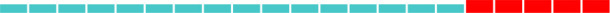
Risk	3.28	2017	2021	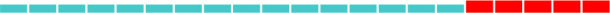
Computed tomography	3.22	2017	2021	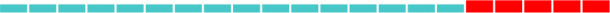
Buyang Huanwu decoction	3.39	2018	2021	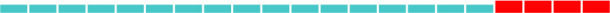

*The beginning of a blue line depicts when an article is published. The beginning of a red segment marks the beginning of a period of burst, whereas the end of the red segment marks the end of the burst period*.

## Discussion

### General Information

Bibliometric indicators such as the number of publications and citations are usually regarded as two major perspectives to evaluate study quality. In general, the number of papers was the yardstick of productivity, and the number of citations without self-citations was applied to measure the impact. It can be seen in Figure 1 that the overall number of publications and citations is on the rise. The amount of literature showed steady growth in the past 20 years, with nearly 5 times more publications in 2021 than in 2001. This reminds us that although major advances in our understanding of BHD over the past several decades, some pivotal questions remain unanswered and research on BHD has still attracted great attention from scholars.

From the perspective of countries and organizations' contributions, we can see that the United States, China, and Japan are the leading countries where BHD research is occurring. Although China ranked second place, all the top 5 organizations were in the other two countries. Centrality is an index to quantify the value of a node that acts as a bridge in the entire network structure. Normally, centrality values >0.1 are considered relatively important nodes. France (0.54) ranked the first, followed by Italy (0.53); National Cancer Institute (the USA) and Yokohama City University (Japan) were the top two organizations of publication outputs, as well as centrality, which reveals they played key parts in BHD research field and bridged in the network of global cooperation. However, from Figures 2A,B, the distribution of countries and organizations were dispersed, the breadth and intensity of major cooperation were not ideal. We can tell that there was less international cooperation and exchange of academic findings. For example, there is a lack of academic collaboration between the top two productive countries—China and the USA; Most collaborating organizations were limited to domestic ones. Considering its rarity, this situation consequently hinders the clinical and fundamental research of BHD. Therefore, it is strongly advised that organizations worldwide should remove academic barriers and enhance cooperation to boost the development of BHD research.

As for authors, W. Marston Linehan contributed the most (53, 7.1%), followed by Laura S Schmidt (39, 5.2%), Mitsuko Furuya (26, 3.5%), Masaya Baba (20, 2.7%), and Berton Zbar (20, 2.7%). It is noteworthy that W. Marston Linehan had the highest citations (5,416), which indicates the most outstanding contributions he had made in the field of BHD during the last 20 years. Dr. Linehan is chief of the Urologic Oncology Branch at the National Cancer Institute, part of the National Institutes of Health in the United States. Since pioneering the research of the genetic basis of kidney cancers, Dr. Linehan and his team have identified six renal cancer genes and five new inherited kidney diseases including BHD. By studying patients and families with kidney cancer (22, 23), he was part of the team which identified *FLCN*, the causative genes for BHD in 2002 (9). He and his colleagues defined the clinical features (27) and provided the basis for the development of new therapeutic strategies for BHD based on understanding the molecular pathway of *FLCN* (28, 29).

According to the journals and co-cited journals in Table 5, the *Plos One* was the most productive one with 19 published papers, and *PNAS* had the highest frequency of co-citations. Nearly all the journals in Table 5 belong to Q1 or Q2. The analysis of the literature sources is helpful to find the core journals in the research field. It can be seen that most cited articles are from high-impact journals, suggesting that the study on BHD is regarded of high value by scholars worldwide.

As shown in Table 6, the most frequently co-cited paper was published by ML Nickerson et.al. in *Cancer Cell* in 2002 (9), which reported *FLCN* gene was identified as the causative gene of BHD. This pioneering finding considerably changed our concepts of the pathogenesis of BHD.

### The Hotspots and Frontiers

Keyword co-occurrence analysis aims at comprehending the distribution and development of research hotspots in a specific field. As demonstrated in Table 7, spontaneous pneumothorax (168), mutation (159), renal cell carcinoma (119), family (83), *folliculin* (83), BHD gene (68), cancer (66), fibrofolliculoma (66), pulmonary cyst (40), diagnosis (39), management (36), and tumor suppressor gene (33) were keywords with high occurrence frequency. Cluster analysis based on keywords eventually generated four clusters. According to these two analyses, we determine the research hotspots and frontiers in the field of BHD. The four main contents are summarized as follows:

#### Genetics and Pathogenesis of BHD

Since its discovery in 2002, *FLCN* remains the only gene involved in BHD to date. Although the exact function of this tumor suppressor gene remains incompletely resolved, it is believed to be involved in the modulation of cell growth, proliferation, and survival through the mammalian target of rapamycin (mTOR) signaling pathway (29, 30). The majority of *FLCN* mutations are protein-truncating, which likely lead to a dysfunction of folliculin (31). Mutations are mainly inherited from an affected parent, but can also occur in people without a family history (32). Although no obvious association relationship between the type or the position of *FLCN* variants and the phenotype of BHD has been established (27), a few studies, have shown several possible genotype–phenotype correlations (33–35). For instance, Toro et al. reported an increased risk of pulmonary cysts in patients carrying mutations in exon 9, as well as more pneumothoraces in patients with variants in exon 9 and 12 (33).

The mechanisms contributing to the formation of pulmonary cyst is not yet completely understood. Evidence has emerged that several dysregulated signaling pathways are involved in the process, including mTOR, AMPK, and impaired cellular adhesion (36). A recent “stretch hypothesis” proposes that cysts could arise because of enhanced cell-cell adhesion and recurrent stretch-induced stress caused by respiration, thus resulting in the expansion of alveolar spaces, especially in lung regions with larger alveolar volume changes (37). This explains the lower lobe predominant pulmonary cysts that occur in up to 90% of patients with BHD. More recent data found that *FLCN*-deficient mesenchymal-lineage cells have defective Wnt pathway activity *via* inhibition of transcription factor TFE3, which suggests that Wnt-dependent cell-cell communication during lung development may lead to lung cyst pathogenesis in BHD (38).

#### Clinical Manifestations of BHD

BHD is characterized by considerable phenotypic heterogeneity. Carriers of *FLCN* gene variants may be asymptomatic or with varying levels of pulmonary, cutaneous, or renal manifestations (24, 39). Other clinical findings include tumors other than renal ones, such as colon polyps. The combination of colon polyps and fibrofolliculomas is reported by Hornstein and Knickenberg in 1975 (1). It is still unclear whether BHD is linked with the elevated risk of colon adenoma and cancer.

##### Pulmonary Manifestations

More than 80% of patients with BHD have multiple bilateral lung cysts (20, 33). There is a 50-times higher risk of pneumothorax for BHD-affected individuals than in the general population (20), and the relapse rate was 75–82% with 3.6 average episodes (33, 40). Changes in atmospheric pressure coming from air travel or diving may lead to an increased risk for developing pneumothorax (41). Lung involvement has no sex predilection and is not related to smoking (33).

Apart from pneumothorax, pulmonary cysts in BHD are usually asymptomatic or only result in mild cough or dyspnoea on exertion. Respiratory function data in BHD have been reported in a few studies (42–45). A large retrospective analysis (*n* = 96) showed that lung function at baseline was not affected except for slightly increased residual volume (RV) and reduced carbon monoxide (*DLco*), and no significant deterioration of lung function during 6-year follow-up (43). In other words, lung involvement in BHD does not bring about the progressive decline of lung function and chronic respiratory insufficiency compared with other cystic lung diseases such as LAM or PLCH.

##### Cutaneous Manifestations

Although cutaneous involvement is common in BHD, potentially as many as 25% of patients may not present with skin lesions (27, 33, 44), and it seems to be less usual (46) and less typical (47) in Asian patients. In general, skin lesions appear as multiple, white-to-flesh colored, smooth, dome-shaped papules. These lesions are mainly on the face, the neck, and sometimes on the trunk or the ears (6). Birt and his colleagues described fibrofolliculomas, trichodiscomas, and acrochordons as a triad of cutaneous lesions that characterize BHD (2). At present, fibrofolliculomas and trichodiscomas are regarded as part of a morphological spectrum. Their identification should raise the suspicion of BHD and prompt further investigations to confirm the diagnosis. Acrochordons, also known as skin tags, are not specific to BHD (6). BHD-associated skin lesions such as angiofibroma may lead to tuberous sclerosis complex (TSC), a differential diagnosis of BHD (48).

##### Renal Manifestations

Renal tumors occur in about 30% of cases (20) at an average age of 50 years (8, 49). It has also been reported in a 20-year-old patient (50). The most threatening manifestation is renal cancer and the risk of developing it is a seven-time increase for BHD patients (20). Renal tumors are multifocal or bilateral in more than 50% of patients with BHD (20, 49). Oncocytomas are the only benign renal tumors of BHD (about 5% of cases). Chromophobe renal cell carcinoma (RCC) accounts for 34% of renal masses in BHD (51). Approximately 50% of kidney neoplasia in BHD are hybrid chromophobe RCC-oncocytoma (51). These two tumors are typical for patients with BHD, accounting for more than 70–80% of *FLCN*-correlated renal tumors, both associated with low malignant potential (10). Other histological subtypes also appear, such as clear cell RCC and papillary RCC, and some mixed patterns (49, 50). Benign renal cysts have been reported in BHD patients and whether they are part of the syndrome is currently uncertain (24, 52).

#### Diagnosis of BHD

Considering its rarity and the broad spectrum of clinical manifestations, the identification of BHD is challenging and might result in a diagnostic delay (40). The symptoms of BHD are often underestimated and improperly assessed, which results in under-diagnosis and underestimation of the number of cases in many regions of the world. Confirmation of BHD diagnosis depends on a combination of clinical manifestations and *FLCN* gene testing. If there is suspicion of BHD, it is recommended to thoroughly search for individual and family history of pulmonary cysts, skin lesions, pneumothorax, and kidney neoplasia, as they are highly predictive of BHD diagnosis. Risky family members should be taken diagnostic radiology exams for lung and kidney involvement. A presymptomatic gene test should also be considered when the familial mutation is known (6). It is worth noting that BHD should be differentiated from other syndromes with similar signs and symptoms, such as LAM or TSC.

#### Management of BHD

Different manifestations of BHD are controlled in different ways. Although there is no danger of malignant transformation, skin lesions associated with psychological burden should not be underestimated. However, therapeutic strategies are limited at present. As no specific therapy for BHD-associated cystic lung disease, pleurodesis should be considered following the initial pneumothorax to decrease the risk of the recurrent episodes (40). Patients should also be discouraged from diving and smoking. Given that BHD rarely result in the decline of lung function and chronic respiratory insufficiency like other cystic lung diseases, it is inadvisable to perform regular follow-up with lung function tests or pulmonary high resolution computed tomography (HRCT) in most patients (10). CT scans, ultrasounds, or MRIs of the kidneys should be conducted regularly on all BHD patients since diagnosis or starting from the age of 20 years (53). Annual renal MRI is the preferred imaging modality, with high sensitivity and no radiation complications (53, 54). As most renal tumors of BHD have low malignant potential and may develop over time, partial nephrectomy is recommended to be postponed until the largest mass reaches 3 cm in diameter. Although mTOR inhibitors, such as everolimus, have shown a beneficial effect in BHD patients (55), more clinical studies are needed to evaluate its efficacy of it in BHD-associated renal tumors.

## Conclusions

This study summarizes the research status of BHD in the past two dacades. BHD-associated publications are increasing over time. Different countries/regions and organizations need to deepen and strengthen their cooperation. The majority of the papers regarding BHD are published in and cited from the international influential journals, indicating that BHD has gained much attention. Some questions remain unanswered, such as the exact function of folliculin and the mechanisms leading to the formation of lung cysts following folliculin dysfunction. Those issues need to be put on the front burner. This study provides assistance for scholars to find core literature and partners in BHD, contributes direction for journals publication, and guidelines identifying research hotspots in this field.

## Data Availability Statement

The original contributions presented in the study are included in the article/supplementary material, further inquiries can be directed to the corresponding author/s.

## Author Contributions

SL and GL conceived the study. SL, KX, and XL collected the data. HX, MH, XX, and JL re-examined the data. SL, LZ, YD, and YL analyzed the data. SL wrote the manuscript. XC and GL reviewed and revised the manuscript. All authors contributed to the article and approved the submitted version.

## Funding

This work was supported by the National Natural Science Foundation of China (Grant No. 82074262).

## Conflict of Interest

The authors declare that the research was conducted in the absence of any commercial or financial relationships that could be construed as a potential conflict of interest.

## Publisher's Note

All claims expressed in this article are solely those of the authors and do not necessarily represent those of their affiliated organizations, or those of the publisher, the editors and the reviewers. Any product that may be evaluated in this article, or claim that may be made by its manufacturer, is not guaranteed or endorsed by the publisher.
